# Inhibition of Bcl-xL overcomes polyploidy resistance and leads to apoptotic cell death in acute myeloid leukemia cells

**DOI:** 10.18632/oncotarget.4306

**Published:** 2015-05-27

**Authors:** Weihua Zhou, Jie Xu, Elise Gelston, Xing Wu, Zhengzhi Zou, Bin Wang, Yunxin Zeng, Hua Wang, Anwen Liu, Lingzhi Xu, Quentin Liu

**Affiliations:** ^1^ State Key Laboratory of Oncology in South China, Cancer Center, Sun Yat-Sen University, Guangzhou, China; ^2^ Department of Oncology, the Second Affiliated Hospital, Nanchang University, Nanchang, China; ^3^ University of Michigan Medical School, Ann Arbor, Michigan, United States; ^4^ MOE Key Laboratory of Laser Life Science and Institute of Laser Life Science, College of Biophotonics, South China Normal University, Guangzhou, China; ^5^ Department of Ultrasound, Union Hospital, Tongji Medical Collage of Huazhong University of Science and Technology, Wuhan, China; ^6^ Department of Hematology, the Third Affiliated Hospital, Sun Yat-Sen University, Guangzhou, China; ^7^ Department of Hematological Oncology, Cancer Center, Sun Yat-Sen University, Guangzhou, China; ^8^ Institute of Cancer Stem Cell, Dalian Medical University, Dalian, China

**Keywords:** mitotic slippage, polyploidy, resistance, Bcl-xL, targeted therapy

## Abstract

Small molecular inhibitors or drugs targeting specific molecular alterations are widely used in clinic cancer therapy. Despite the success of targeted therapy, the development of drug resistance remains a challenging problem. Identifying drug resistance mechanisms for targeted therapy is an area of intense investigation, and recent evidence indicates that cellular polyploidy may be involved. Here, we demonstrate that the cell cycle kinase inhibitor, Oxindole-1 (Ox-1), induces mitotic slippage, causing resistant polyploidy in acute myeloid leukemia (AML) cells. Indeed, Ox-1 decreases the kinase activity of CDK1 (CDC2)/cyclin B1, leading to inhibition of Bcl-xL phosphorylation and subsequent resistance to apoptosis. Addition of ABT-263, a Bcl-2 family inhibitor, to Ox-1, or the other polyploidy-inducer, ZM447439 (ZM), produces a synergistic loss of cell viability with greater sustained tumor growth inhibition in AML cell lines and primary AML blasts. Furthermore, genetic knockdown of Bcl-xL, but not Bcl-2, exhibited synergistic inhibition of cell growth in combination with Ox-1 or ZM. These data demonstrate that Bcl-xL is a key factor in polyploidization resistance in AML, and that suppression of Bcl-xL by ABT-263, or siRNAs, may hold therapeutic utility in drug-resistant polyploid AML cells.

## INTRODUCTION

Genomic instability, a hallmark of transformed cells, is thought to drive tumorigenesis by favoring the generation of aggressive tumor cells with a reduced propensity for apoptosis [1]. One mechanism of genomic instability involves a transient phase of polyploidization followed by asymmetric cell divisions and/or chromosome loss, resulting in aneuploidization and chromosomal instability [2].

Polyploidy, a state in which cells possess more than two complete sets of homologous chromosomes, usually consists of an even number of sets with four (tetraploidy) being the most common [3, 4]. Tetraploid cells are generated through a variety of mechanisms, including cytokinesis failure and viral-induced cell fusion [3]. Cells can also become tetraploidy after prolonged mitotic arrest by spindle assembly checkpoint (SAC), which functions to arrest mitotic cells with defects that prevent normal kinetochore–microtubule attachment [5]. If SAC is not satisfied, cells exit mitosis and enter the next G_1_ as tetraploid cells in a process known as mitotic “slippage”, which requires the ubiquitylation and proteolysis of cyclin B1 [3, 6], a regulatory subunit of cyclin-dependent kinase 1 (CDK1, also known as CDC2). Mitotic slippage occurs without karyokinesis and results in polyploidy cells with a single large nucleus, unlike cytokinesis failure and cell fusion, which give rise to binucleate cells [3]. Due to a weakened SAC, cells may slip out of mitotic arrest before they die; thus, mitotic slippage protects cells from death and the resulting tetraploidy could pose a barrier to chemotherapy against malignancy [7–9].

Indeed, recent studies support a link between polyploidy and resistance to chemotherapy: polyploid giant cancer cells were shown to be resistant to cisplatin treatment [10], and cisplatin treatment led to the induction of 4N tetraploidy in HCT116 cisplatin-resistant clones [11]. Likewise, small molecular inhibitors generate polyploid cancer cells [12–14], and induce apoptosis secondary to polyploidization [13, 15]. However, polyploidization is not an absolute commitment to apoptosis because polyploid cells can spawn viable progeny [16], possibly leading to drug resistance or more aggressive secondary tumors. For example, preexisting cellular polyploidy had intrinsic resistance to CDK2 inhibitors [17], while breast cancer cell polyploidy secondary to small-molecule inhibitor BMS-777607 demonstrated increased resistance to chemotherapeutics [18].

Previous studies suggest two mechanisms by which polyploidy avoids apoptosis in chemotherapy. The first one is by up-regulation of pro-survival members from the Bcl-2 family proteins. For example, overexpression of Bcl-xL protects against mitochondrial outer membrane permeabilization (MOMP) and subsequently leads to polyploidy resistance promoted by Aurora B inhibitor [19] ; furthermore, elevated Mcl-1 expression increased mitotic slippage and attenuated apoptosis in the polyploidy cells caused by anti-tubulin chemotherapeutics [20]. The second mechanism is by slippage-induced inhibition of CDK1 (CDC2)/cyclin B1 kinase activity. CDK1 (CDC2)/cyclin B1 phosphorylates and inactivates the anti-apoptotic members, including Bcl-2, Bcl-xL, and Mcl-1 [8, 20, 21]; consequently, slippage-induced inhibition of CDK1 (CDC2)/cyclin B1 kinase activity leads to reduction of apoptosis [8, 9]. Better understanding of the mechanisms by which polyploid cells evade apoptosis will improve efficacy of small molecular inhibitors and minimize the danger posed by post-treatment, genetically reshuffled tumor cells.

Here, we identify that Bcl-xL dephosphorylation, which is resulted from mitotic slippage and the resulting inhibition of CDK1 (CDC2)/cyclin B1 kinase activity, is responsible for the viability of resistant small molecular inhibitor-induced polyploid cells. We show that inhibition of Bcl-xL by small molecular inhibitor, ABT-263, or by siRNAs, triggers the rapid demise of polyploid cells in AML cell lines and primary bone marrow blasts, but not in normal, non-transformed cells. Our data suggest that combining polyploidy-inducing small molecular inhibitors with agents targeting Bcl-xL could be a promising strategy for AML therapy.

## RESULTS

### Ox-1 treatment produces resistant polyploidy cells in AML cell line

**Figure 1:**
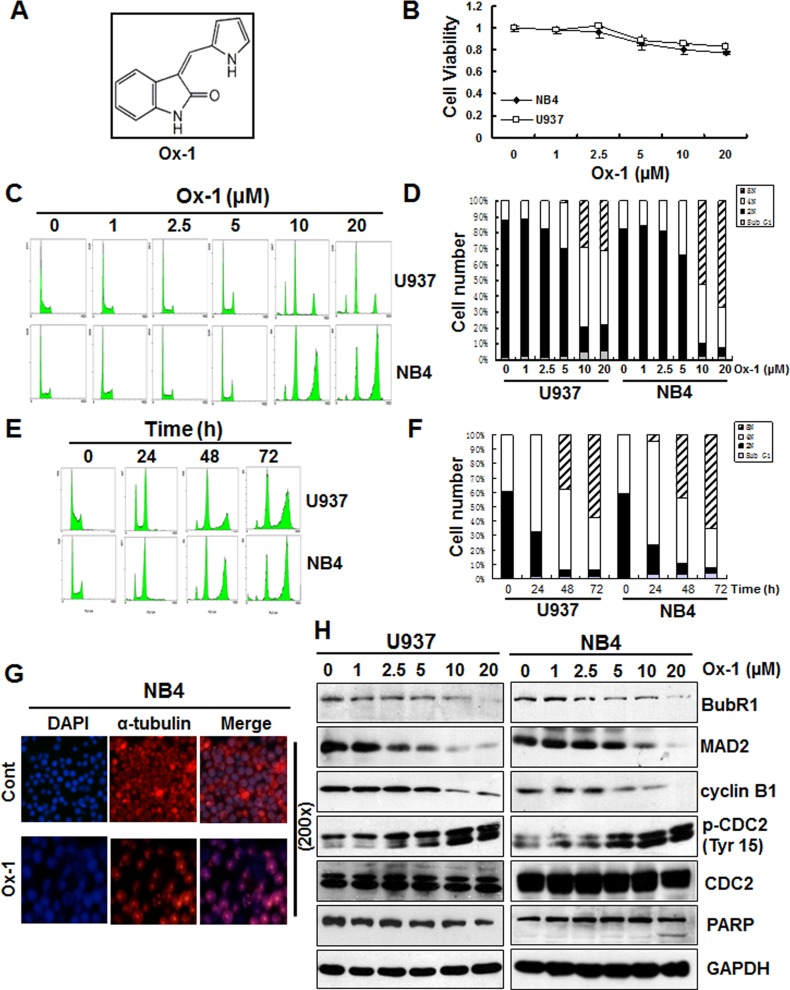
Ox-1 treatment produces resistant polyploidy through mitotic slippage in AML cell lines **A.** Chemical Structure of 3-(1H-Pyrrol-2-ylmethylene)-1,3-dihydroindol-2-one (Ox-1). **B.** Ox-1 minimally inhibits cell growth of NB4 and U937 AML cell lines. Cells (5000 cells/well) were treated with DMSO or different doses of Ox-1 for 48h. MTT assay was performed to detect the cell viability as described in “Materials and Methods”. **C.** & **D.** Ox-1 treatment induces resistant polyploid cells in a dose-dependent manner. U937 and NB4 cells were treated with DMSO or different doses of Ox-1 for 48h. Cells were then collected for propidium iodide staining and flow cytometry. Percentage of the cell population of Sub G_1_, 2N, 4N, 8N, in U937 and NB4 were shown. **E.** & **F.** Ox-1 treatment induces resistant polyploid cells in a time-dependent manner. U937 and NB4 cells were treated with the same concentration of Ox-1(10 μM) for different time. Cells were then collected for propidium iodide staining and flow cytometry. Percentage of the cell population of Sub G_1_, 2N, 4N, 8N, in U937 and NB4 were shown. **G.** Ox-1 induced mono-polar polyploidy in AML cells. Cells were treated with DMSO or Ox-1 (10 μM) for 48h and then collected for immunofluorescence staining. **H.** Ox-1 treatment induces SAC inactivation and leads to mitotic slippage. DMSO or different doses of Ox-1 were administrated to NB4 and U937 cells for 48h and then cells were collected for the immunoblotting by using the indicated antibodies.

Oxindole-1 (Ox-1) (Figure 1A), a potent and selective inhibitor of vascular endothelial growth factor (VEGF) receptor tyrosine kinase Flk-1, also inhibits CDK4/cyclin D1 enzyme (Figure S1A) [22], which plays a crucial role in regulating G_1_/S transition. Aberrant activity of CDK4/cyclin D1 has been implicated in several cancers, and blocking CDK4/cyclin D1 activity with Ox-1 was previously shown to be an effective anticancer therapy for pRb^+^ solid tumors [22]. In the present study, we noticed that Ox-1 minimally inhibits cell growth of NB4 and U937 AML cell lines (Figure 1B). Flow cytometry analysis confirms that Ox-1 hardly induces apoptosis in AML cell lines as manifested by the minimal Sub G_1_ peak (Figure 1C–1F). Furthermore, we fail to detect any effects of Ox-1 on G_1_/S transition, though Ox-1 treatment induces appearance of polyploid cells in a dose- (Figure 1C and 1D) and time-dependent manner (Figure 1E and 1F).

The minimal concentration of Ox-1 required to induce polyploidy is 5 μM for both U937 and NB4 cells. At this concentration, 30.1% (29% 4N; 1% 8N) of U937 and 30.6% (30.6% 4N; 0% 8N) of NB4 cells underwent polyploidy. The percentages were significantly higher when Ox-1 was used at 10 μM (49.8% 4N and 29.6% 8N in U937; 37% 4N and 52% 8N in NB4) to 20 μM (45% 4N and 31% 8N in U937; 25.2% 4N and 66.8% 8N in NB4) (Figure 1D). Time-dependent polyploidy is shown in Figure 1E and 1F. Polyploidy was seen as early as 24h after 10 μM Ox-1 treatment. More than 80% of cells underwent polyploidy in U937 (34.1% 4N; 53.3% 8N), and NB4 (25.1% 4N; 60.3% 8N) cells after 72h of treatment.

Our results indicate a delayed progression of cellular mitosis in the presence of Ox-1, which prompts us to consider affected microtubule dynamics. We therefore conducted immunofluorescent staining to evaluate α-tubulin in spindle assembly. Compared with untreated control cells, NB4 cells treated with Ox-1 show a single large nucleus with severely disorganized mitoses marked by mono-polar spindle assembly, indicating the absence of karyokinesis (Figure 1G).

### Ox-1 treatment induces SAC inactivation and leads to subsequent mitotic slippage

SAC normally functions to delay progression into anaphase until all chromosomes attain bipolar attachment to the mitotic spindle by inhibiting Cdc20, a co-factor of the ubiquitin ligase anaphase-promoting complex/cyclosome (APC/C). An activated SAC binds to Cdc20 via its proteins, MAD2 and BubR1, to inhibit the ability of Cdc20 to activate the APC/C-mediated proteolysis of cyclin B1, whereas unsatisfied SAC fails to sequester Cdc20 and ultimately activates APC/C, leading to the degradation of cyclin B1 [23, 24].

Our present study supports that Ox-1 treatment reduces the levels of SAC proteins, MAD2 and BubR1 (Figure 1H); IP pull-down assay further indicated that MAD2 and BubR1 were decreased in Cdc20 immunoprecipitates when cells were treated with dose-dependent Ox-1 (Figure S1B). These data indicate that Ox-1 treatment promotes the inactivation of SAC and leads to the decrease of Cdc20 inhibition. Consequently, cyclin B1 was gradually degraded in a dose-dependent way, followed by concurrently inhibition of CDK1 (CDC2) kinase activity as manifested by the increase of the inhibitory phosphorylation of Tyr15, while pro-apoptotic protein cleavage PARP fails to rise (Figure 1H). Therefore, it appears that Ox-1 treatment permits cell entry into anaphase despite not satisfying SAC, contributing to mitotic slippage and resistance to apoptosis.

### Bcl-xL phosphorylation (Ser62) is decreased upon Ox-1 treatment

**Figure 2:**
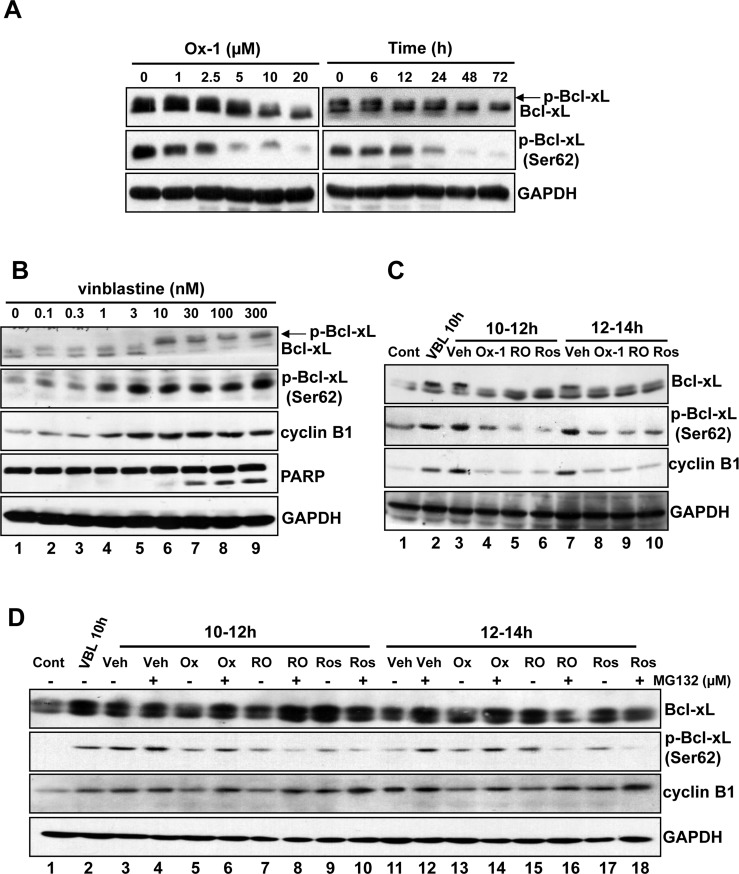
Bcl-xL phosphorylation (Ser62) is inhibited by Ox-1 treatment **A.** Ox-1 inhibits Bcl-xL phosphorylation in a dose- and time-dependent way. NB4 cells were treated with Ox-1 for different doses or time and then cells were collected for the immunoblotting by using the indicated antibodies. **B.** Vinblastine induces Bcl-xL phosphorylation and apoptosis. NB4 cells were treated with vinblastine at the indicated concentrations for 16h, and extracts were prepared for immunoblotting for the indicated proteins. Arrows indicate phosphorylated forms of Bcl-xL. **C.** Ox-1 treatment blocks Bcl-xL phosphorylation (Ser62) during mitotic arrest. NB4 cells were synchronized at the G_1_/S boundary by a double thymidine block and were treated with 30 nM vinblastine (VBL) 1h after release. Cells were harvested at 10h after release or were treated with 25 μM Ros, 10 μM RO, or the DMSO vehicle (Veh) for 2h during the periods from 10 to 12h, and 12 to 14h after release. An untreated dish of cells harvested immediately after release from the double thymidine block (Con, 0h) was included as a control. Whole-cell extracts were prepared and immunoblotting for the indicated proteins. **D.** Ox-1 but not the CDK1 inhibitors reversed the inhibition of Bcl-xL phosphorylation when the proteasome is inhibited. NB4 cells were synchronized at the G_1_/S boundary by a double thymidine block and were treated with 30 nM VBL 1h after release. Cells were harvested 10h after release or were incubated for 2h during 10 to 12h after release, or from 12 to 14h after release with 25 μM Ros, 10 μM RO, or DMSO in the presence or absence of the proteasome inhibitor MG132 (25 μM). MG132 was added 20 min prior to the addition of Ox-1 or the CDK1 inhibitors. Immunoblotting for the indicated proteins was performed.

CDK1 (CDC2)/cyclin B1 kinase complex is reported to be responsible for microtubule inhibitor (MTI)-induced Bcl-xL/Bcl-2 phosphorylation and inactivation of their anti-apoptotic effects, leading to cell death [8]. In particular, phosphorylation of Bcl-xL (Ser62) plays a crucial role in MTI-induced cell death [8]. Due to the suppression of CDK1 (CDC2)/cyclin B1 activity upon the treatment with Ox-1 (Figure 1H), we detected the expression of Bcl-xL and p-Bcl-xL (Ser62) by treatment with Ox-1 in NB4 cells. Indeed, Ox-1 reduced the phosphorylation of Bcl-xL and expression of p-Ser62 in a dose- and time-dependent manner (Figure 2A). To further demonstrate that, we treated NB4 cells with increasing concentrations of vinblastine, which shows a dose-dependent increase of Bcl-xL phosphorylation and p-Bcl-xL (Ser62) expression, with a dose dependency of cyclin B1 and cleavage PARP strikingly similar to the level of Bcl-xL phosphorylation (Figure 2B).

We next tested whether Ox-1 could reverse vinblastine-induced phosphorylation of Bcl-xL. For this purpose, we selected two validated CDK1 (CDC2) inhibitors, Roscovitine (Ros) [25] and RO-3306 (RO) [26], as positive controls. Cells were synchronized with a double thymidine block and treated with vinblastine 1h after release from G_1_/S phase. The CDK1 (CDC2) inhibitors, Ox-1, or a dimethyl sulfoxide (DMSO) vehicle, were added for 2h at 10h or 12h following G_1_/S release in the presence of vinblastine. This experimental strategy is schematized in Figure S2A. Extracts were made and subjected to immunoblotting for Bcl-xL, p-Bcl-xL (Ser62), and cyclin B1 (Figure 2B). Lane 1 shows the baseline, i.e. blots of cell extracts at time of the second release (0h). Evidenced by comparison of Lane 1 and Lane 2 in Figure 2C, vinblastine induces a marked increase in Bcl-xL phosphorylation at 10h post-G_1_/S release as well as during the interval from 10h to 12h post-G_1_/S release (Lane 1 and Lane 3), as expected. Ox-1 and the CDK1 (CDC2) inhibitors all blocked this increase (Figure 2C; comparison of Lane 3 with Lanes 4-6). Effects are similar at 12h to 14h post-G_1_/S release in the presence of vinblastine (Figure 2C; Lanes 7-10). These data indicate that Ox-1, via reducing the activity of CDK1 (CDC2)/cyclin B1, inhibits vinblastine-induced Bcl-xL phosphorylation at Ser62.

### Ox-1 inhibits vinblastine-induced Bcl-xL phosphorylation through mitotic slippage

We next determine whether Ox-1 affects Bcl-xL phosphorylation through mitotic slippage, which can lead to the decrease of CDK1 (CDC2)/cyclin B1 activity, or direct inhibition of CDK1 (CDC2) kinase. Cells were synchronized with a double thymidine block and treated with either a vehicle or vinblastine at 1h post-release; MG132 (25 μM), which inhibits the mitotic slippage by suppressing the degradation of cyclin B1, was then added 20 min prior to the addition of the vehicle, CDK1 (CDC2) inhibitors, or Ox-1, at 10h post-release, with cells harvested at 12h and 14h post-release. This experimental strategy is schematized in Figure S2B. As shown in Figure 2D, MG132 reverses Ox-1-induced-dephosphorylation of Bcl-xL, and increases the expression of Ser62 and cyclin B1 at 10-12h post-release (Figure 2D; comparison of Lane 5 with 6) and 12-14h post-release (Figure 2D; comparison of Lane 13 with 14). MG132 fails to reverse similar effects caused by CDK1 (CDC2) inhibitors (Figure 2D; comparisons of Lane 7 with 8; Lane 9 with 10; Lane 15 with 16; Lane 17 with 18). Thus, we conclude that small molecular inhibitor Ox-1 inhibits vinblastine-induced Bcl-xL phosphorylation through mitotic slippage rather than direct inhibition of CDK1 (CDC2) kinase.

### Bcl-xL inhibition elicits synergistic cytotoxicity with Ox-1

**Figure 3:**
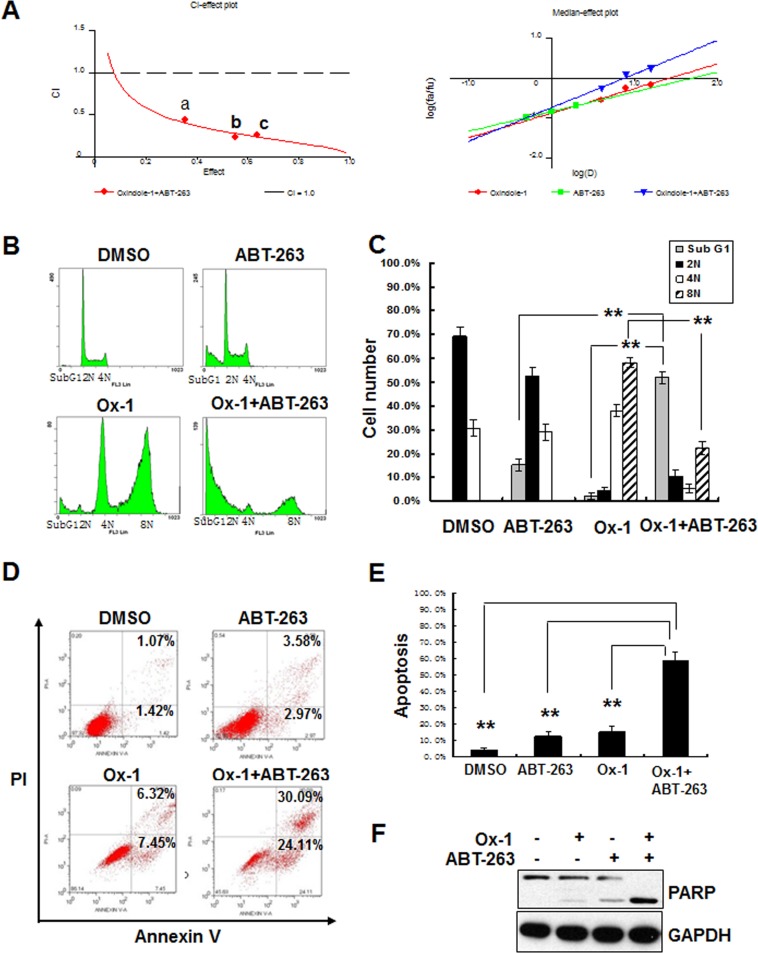
Ox-1 in combination with ABT-263 elicits synergistic cytotoxicity **A.** Ox-1 elicits synergy with ABT-263. CI-effect plots and medianeffect plots were generated using CalcuSyn software. The points a, b, and c represent CI values for the combinations 5, 10, and 20 μM Ox-1 with 0.5, 1, and 2 μM ABT-263 in a constant ratio, respectively. **B.** & **C.** ABT-263 blocks Ox-1-induced polyploidy. NB4 cells were incubated with DMSO, Ox-1 (10 μM), and/or ABT-263 (1 μM) for 48h; cells were then collected, fixed with ice-cold 70% ethanol overnight, and collected for propidium iodide staining and flow cytometry. Shown are mean ± SD, **P < 0.01. **D.**–**F.** ABT-263 triggers apoptosis in Ox-1-induced polyploidy cells. NB4 cells were treated with DMSO, Ox-1 (10 μM), and/or ABT-263 (1 μM) for 48h; cells were then harvested for annexin V assay (**D.** & **E.**) or immunoblotting for indicated proteins (**F.**). Data summarized three independent experiments. Shown are mean ± SD, **P < 0.01.

To identify agents that could be combined therapeutically with Ox-1, we tested cell viability effects of Ox-1 in combination with several established and experimental cancer therapeutics often used in AML. We did not find any synergistic cytotoxicity of Ox-1 with these agents (data not shown). We postulated that Ox-1-induced polyploid cells may account for the lack of synergy with other cytotoxic chemotherapy agents [18], and so addition of orally bioavailable ABT-263, a small-molecule BH3 mimetic that inhibits Bcl-xL, Bcl-2, and Bcl-w [9, 21], was tested to enhance the cytotoxic activity of Ox-1. NB4 cells were cultured with combinations of these two drugs at different doses but in a constant ratio (Ox-1 to ABT-263: 5 μM to 0.5 μM,10 μM to 1 μM, and 20 μM to 2 μM, respectively) for 48h. Both CalcuSyn software [27, 28] and Jin's formula [29] were used to determine the synergy of the two agents. The combination of 5 μM Ox-1 with 0.5 μM ABT-263 in NB4 cells inhibited growth by 35.39%, compared with monotherapy of Ox-1(22.44%) or ABT-263 (9.54%), indicating synergism (CI = 0.441; Q = 1.19). Escalating doses, i.e. co-treatment with 10 μM Ox-1 and 1 μM ABT-263 (CI = 0.231; Q = 1.24) or 20 μM Ox-1 and 2 μM ABT-263 (CI = 0.260; Q = 1.25), show synergetic effects in NB4 cells (Figure 3A).

Cell cycle distribution further shows that Ox-1 mono-therapy induces huge polyploid cells (35.1% 4N and 55.7% 8N) that are blocked significantly in combination with ABT-263 (7.1% 4N and 25.3% 8N) (Figure 3B and 3C). ABT-263 meanwhile triggers rapid apoptosis in polyploid cells as seen by an increase in Sub G_1_ from 3.9% (Ox-1 alone) to 49.8% (combination of Ox-1 and ABT-263) (Figure 3B and 3C). Furthermore, Ox-1 combined with ABT-263 induces significant apoptotic cell death in an Annexin V-FITC assay (Figure 3D and 3E) as well as significant increase of the pro-apoptotic cleaved PAPR and the phosphorylation of Bcl-xL (Ser62), with no effects on the expression of MAD2 and BubR1 (Figure 3F).

**Figure 4:**
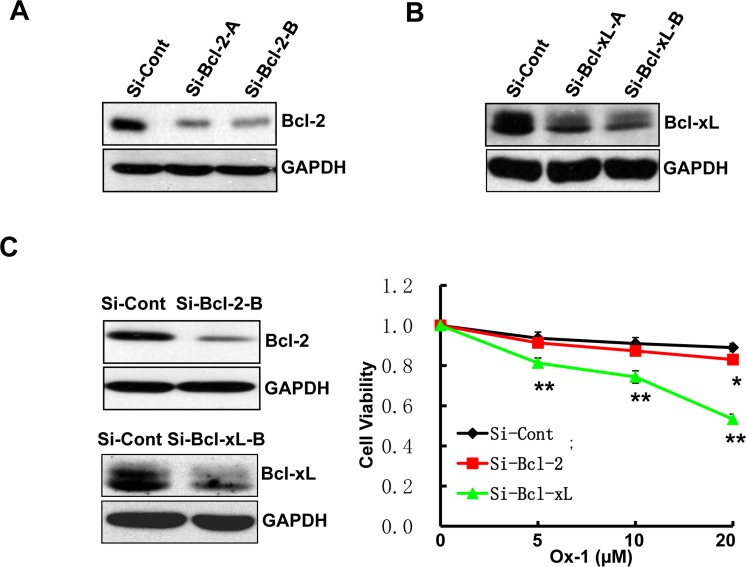
Ox-1 in combination with Bcl-xL silencing elicits synergistic cytotoxicity **A.** & **B.** Two target-specific siRNAs can silence Bcl-2 and Bcl-xL, respectively. **C.** Bcl-xL knockdown enhances the cytotoxic effect of Ox-1 in AML cell line. NB4 cells were transfected with Bcl-xL or Bcl-2 siRNA, or siRNA control for 24h, and then treated with different doses of Ox-1 (5, 10, 20 μM, respectively) for 48h. The numbers of viable cells were measured by trypan blue dye exclusion assay. Shown are mean ± SD, * *P* < 0.05; ** *P* < 0.01 (compared with control).

ABT-263 simultaneously inhibits Bcl-2, Bcl-xL, and Bcl-w; two of which (Bcl-2 and Bcl-xL) are co-expressed in many human cancer cells [30, 31]. Accordingly, we applied siRNAs to determine which of the two ABT-263 targets, when inhibited, would phenocopy the synergistic activity observed for ABT-263 in combination with Ox-1. We designed two target-specific siRNAs for both Bcl-2 (Figure 4A) and Bcl-xL (Figure 4B), with the second siRNA for both of them showing the significant inhibition and being used in the following experiments. Subsequent results showed that Bcl-xL silencing alone exhibited synergistic inhibition of cell growth in combination with Ox-1 (Figure 4C), suggesting that ABT-263 inhibition of Bcl-xL is responsible for the synergistic cytotoxicity with Ox-1.

### Bcl-xL inhibition elicits synergistic cytotoxicity with ZM447439

**Figure 5:**
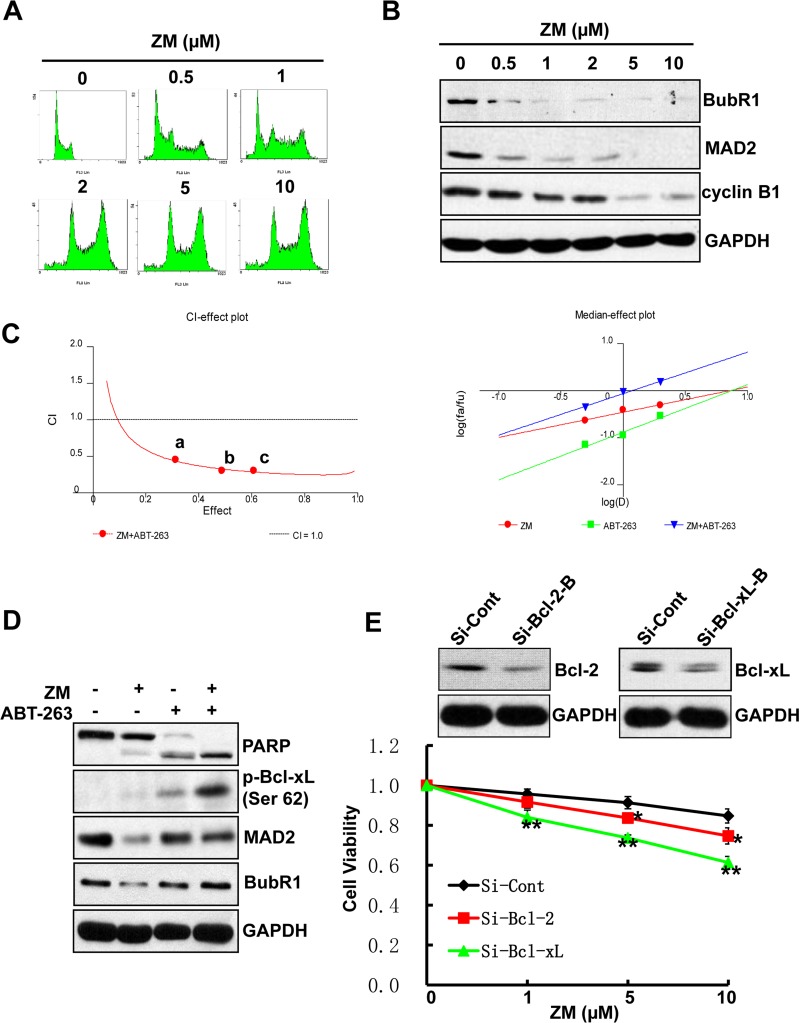
ABT-263 elicits synergistic cytotoxicity with ZM **A.** ZM induces polyploidy in AML cells. NB4 cells were incubated in fresh media containing different doses of ZM (0.5, 1, 2, 5, and 10 μM) or DMSO for 48h. The cell-cycle stage and apoptosis were assessed by propidium iodide staining and flow cytometry. **B.** ZM induces mitotic slippage in AML cells. NB4 cells were incubated with different doses of ZM (0.5, 1, 2, 5 and 10 μM) or DMSO for 48h; Whole-cell extracts was subjected to immunoblotting by using indicated antibodies. **C.** ZM and ABT-263 synergistically inhibit cell proliferation in AML cells. CI-effect plots and medianeffect plots were generated using CalcuSyn software. The points a, b, and c represent CI values for the combinations 0.5, 1, and 2 μM ZM with 0.5, 1, and 2 μM ABT-263 in a constant ratio, respectively. **D.** ABT-263 triggers apoptosis in Ox-1-induced polyploidy cells. NB4 cells were treated with DMSO, Ox-1 (10 μM), and/or ABT-263 (1 μM) for 48h; cells were then harvested for immunoblotting with indicated antibodies. **E.** Bcl-xL RNAi can enhance the cytotoxic effect of ZM in AML cells. NB4 cells were transfected with siRNA Bcl-xL, Bcl-2, or siRNA control for 24h, and then treated with different doses of ZM (0.5, 1, 2 μM, respectively) for 48h. The numbers of viable cells were measured by trypan blue dye exclusion assay. Shown are mean ± SD, * *P* < 0.05; ** *P* < 0.01 (compared with control).

We confirm the above findings with ZM447439 (ZM), an Aurora selective ATP-competitive inhibitor [32] that induces polyploidy in a dose-dependent manner in AML cell lines (Figure 5A). ZM treatment also results in reduced levels of SAC proteins, MAD2 and BubR1, as well as cyclin B1 protein, indicating the occurrence of mitotic slippage (Figure 5B). Both CalcuSyn software and Jin's formula show that ZM, like Ox-1, has synergistic effects with ABT-263 in AML cell lines (Figure 5C). In addition, Bcl-xL silencing in combination with ZM exhibits significantly more synergistic inhibition of cell growth than the combination with Bcl-2 silencing and control. Furthermore, we found that ABT-263 also triggered robust apoptosis in polyploidy cells caused by the myosin II inhibitor blebbistatin [19] (Figure S3A & S3B).

To further demonstrate that polyploid phenotype renders cell survival dependent on the anti-apoptotic activity of Bcl-xL, we detected the expression of Bcl-xL in multiple AML cell lines. We found that Bcl-xL showed moderate expression in U937 and NB4 cell lines, but much higher in Kasumi-1 and KG1a AML cell lines (Figure S4A), which both show high expression of CD34 surface antigen and are insensitive to Daunorubicin (DNR) in our previous study [33]. Furthermore, the MTT assay indicated that ABT-263 exhibited significantly more synergistic inhibition of cell growth with Ox-1 in Kasumi-1 and KG1a cell lines, compared with that in U937 and NB4 cell lines (Figure S4B), indicating that more malignant AML cell lines show higher sensitivity to ABT-263. Hence, inhibition of Bcl-xL with its inhibitor ABT-263 serves as a promising strategy to overcome polyploid resistance in AML.

### ABT-263 enhances the efficacy of small molecular inhibitors in primary AML blasts but not in normal primary bone marrow cells

**Figure 6:**
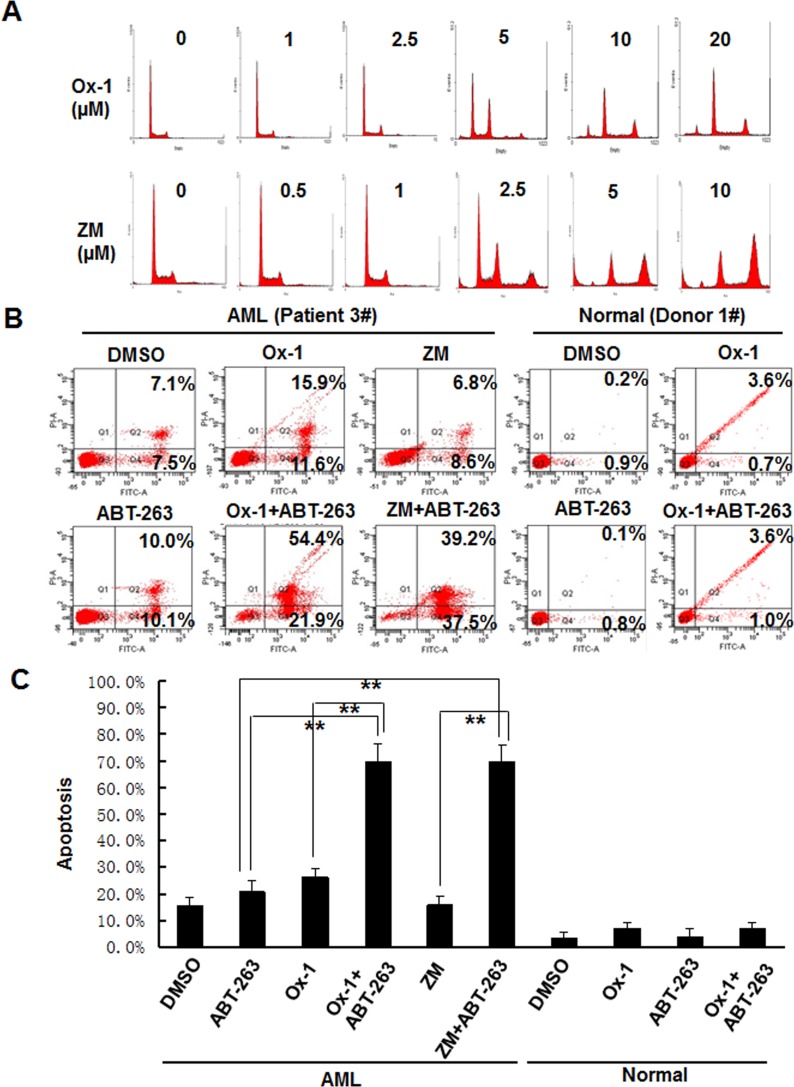
ABT-263 enhances the efficacy of small molecular inhibitors in primary AML blasts but not in normal primary bone marrow cells **A.** Blasts from AML patients were incubated with DMSO, or different doses of Ox-1 or ZM for 48h. Cells were then collected for propidium iodide staining and flow cytometry. **B.** Blasts from AML patient were incubated with indicated doses of Ox-1 (10 μM), ZM (5 μM), ABT-263 (1 μM), or DMSO for 48h; Blasts from the normal donor were incubated with Ox-1 (10 μM), ABT-263 (1 μM), or DMSO for 48h before stained with annexin V assay. Detailed apoptosis analyses performed in primary AML blasts and normal blasts are shown (Bottom). Shown are mean ± SD, ** *P* < 0.01 (compared with control).

Lastly, we asked whether Ox-1 and ZM could induce resistant polyploidy in primary leukemia blasts, and whether Bcl-2 family inhibitor ATB-263 could induce apoptosis in those polyploid cells. Exposure of primary leukemia blasts to Ox-1 and ZM for 48h produces marked polyploidy in a dose-dependent manner (Figure 6A). After incubation of primary leukemia blasts with DMSO, Ox-1, or ZM, and/or ABT-263 for 24h, Annexin V-FITC staining shows that Ox-1 or ZM alone induces minimal apoptosis, but in combination with ABT-263 significantly enhances cell death via apoptosis in primary blasts. In contrast, combining Ox-1 with ABT-263 does not produce synergistic cytotoxicity in normal bone marrow cells (Figure 6B). Detailed apoptosis analyses performed in primary AML blasts and normal blasts are shown (Figure 6C).

## DISCUSSION

Small molecular inhibitors and drugs targeting specific molecular alterations, alone or in combination with standard chemotherapies, are widely used in clinic. Despite the success of these new compounds, their main limitation is the development of resistance. Alteration in the activity and expression of proteins have already been linked to the onset of resistance [34], but recent evidence indicates that polyploidization plays a role as well [17, 18]. In this study, we find that VEGF receptor tyrosine kinase Flk-1 and CDK4/cyclin D1 enzyme inhibitor, Ox-1 (Figure 1A; Figure S1A), minimally inhibits cell growth in AML cell lines (Figure 1B), and minimally induces cell apoptosis by cell cycle distribution, and in fact generates resistant polyploid cells in a dose- and time-dependent manner (Figure 1C–1F). The mechanism by which polypoloid cells remain viable is unclear and merits further investigation.

Polyploidy, often tetraploidy, commonly originates from cell fusion or cytokinesis [3] though also occurs through mitotic slippage induced by SAC-dissatisfaction [7, 35]. Cell fate after mitotic arrest depends on two competing networks: cyclin B1 degradation and the generation of death signals that activate caspases [7, 36]. Slippage occurs if cyclin B1 levels fall below the mitotic exit threshold before the generation of sufficient death signals, whereas cell death occurs if sufficient death signals accumulate before cyclin B1 is adequately degraded. Accordingly, slippage and apoptosis can be viewed as two competing pathways. Here, we note that Ox-1 treatment induces decreases in SAC proteins, MAD2 and BubR1 (Figure 1H), and their binding with Cdc20 (Figure S1B). Furthermore, cyclin B1 was gradually degraded in a dose-dependent way, followed by the concurrent decrease of CDK1 (CDC2) activity, indicating the occurrence of mitotic slippage. Mitotic slippage is further supported by the failure of cleavage PARP levels to increase following Ox-1 treatment (Figure 1H). At this time, it is not clear how Ox-1-induced polyploid cells escape apoptosis and would be worth studying in the future.

Recent studies reported that fates of mitotic slippage-induced polyploid cells depend on the actions of CDK1 (CDC2)/cyclin B1 on proteins that directly regulate apoptosis. CDK1 (CDC2)/cyclin B1 phosphorylates and inactivates anti-apoptotic members of the Bcl-2 protein family (Bcl-2, Bcl-xL, and Mcl-1) [5, 8, 20], which are necessary for mitochondrial apoptosis and caspase activation. Mitotic slippage can lead to the inhibition of CDK1 (CDC2)/cyclin B1 activity, ultimately resulting in the dephosphorylation of Bcl-2 family members and subsequent reduction in apoptosis [9]. Here, we demonstrated that Ox-1 suppressed the phosphorylation of Bcl-xL and reduced the expression of p-Ser62 in a dose- and time-dependent way (Figure 2A). Vinblastine normally facilitates phosphorylation of Bcl-xL at Ser62, resulting in disabled anti-apoptotic activity of Bcl-xL [8], which is supported by our findings (Figure 2B). In addition, we show that Ox-1, like our two tested CDK1 (CDC2) inhibitors, inhibits vinblastine-induced Bcl-xL phosphorylation and decreases the expression of p-Ser62 (Figure 2C). We show that these Ox-1-induced changes, unlike those produced by the two CDK1 (CDC2) inhibitors, are rescued by MG132 (Figure 2D); thus, Ox-1 promotes the dephosphorylation of Bcl-xL via mitotic slippage rather than direct inhibition of CDK1 kinase.

To rid AML cell lines of remaining resistant polyploid cells induced by Ox-1, we tried combination therapy with ABT-263, a Bcl-2 family inhibitor. Both CalcuSyn software and Jin's formula show that ABT-263 has synergistic cytotoxicity with Ox-1 (Figure 3A). ABT-263 reverses Ox-1-mediated polyploid cells and induces rapid apoptotic cell death (Figure 3B–3F). However, ABT-263 has two main targets and we show that Bcl-xL silencing, not Bcl-2 silencing, is responsible for ABT-263 synergy with Ox-1 (Figure 4C) and may eventually play a crucial role in combating Ox-1-drug resistance. We then asked ourselves whether ABT-263 or Bcl-xL silencing could be effective against drug resistance seen with other polyploidy-inducers such as ZM447439, an Aurora selective ATP-competitive inhibitor [32] that induces polyploidy through mitotic slippage in AML cell lines (Figure 5A and 5B). We find that ABT-263, via Bcl-xL inhibition, elicits synergistic effects when combined with ZM (Figure 5C–5E). Notably, ABT-263 induced robust apoptosis in polyploidy cells made by the myosin II inhibitor blebbistatin [19] (Figure S3A and S3B). Our following MTT assay further indicated that ABT-263 showed more synergistic inhibition of cell growth with Ox-1 in Kasumi-1 and KG1a cell lines, which are both characterized by high expression of CD34 surface antigen [33] and Bcl-xL (Figure S4A and S4B). Hence, Bcl-xL is a target for polyploidy resistance and the cells with overexpression of Bcl-xL are more sensitive to Bcl-xL inhibition, supporting that ABT-263 serves as a promising method of reversing polyploidy drug resistance seen in more malignant AML cells.

ABT-263 has already proven efficacious in solid tumors in a Phase 1 clinical study [37], but it has not yet been tested in hematologic malignancies. In primary AML blasts, we find that Ox-1 and ZM produce significant numbers of polyploid cells (Figure 6A). Furthermore, administration of Ox-1 or ZM alone induces minimal apoptosis in AML blasts, whereas combination regimens of Ox-1 or ZM plus ABT-263 produce significant apoptotic cell death (Figure 6B and 6C). It is worth noting that Ox-1 treatment combined with ABT-263 does not produce synergistic cytotoxicity in normal bone marrow cells (Figure 6B and 6C). We believe that ABT-263 could be a potential therapeutic to enhance the cytotoxicity of polyploidy inducers in AML cells while sparing normal bone marrow.

In summary, we demonstrate the pro-survival function of Bcl-xL in cancer polyploid cells induced by small molecular inhibitors. Furthermore, combination of Bcl-xL inhibitor and polyploid inducers produces synergistic anti-proliferative activity *in vitro* and enhanced efficacy *in vivo*. Moreover, these data support a potential therapeutic application of ABT-263 that specifically exploits and targets polyploid phenotype in malignancy.

## MATERIALS AND METHODS

### Reagent and cell culture

Ox-1 (Santa Cruz), ZM (ACC), blebbistatin (APEXBIO), ABT-263 (Selleckchem), Roscovitine (Santa Cruz), RO-3306 (Santa Cruz), were dissolved in dimethyl sulfoxide (DMSO) and stored at −20°C. Human NB4 and U937 cell lines were obtained from the American Type Culture Collection (ATCC) and grown in RPMI 1640 medium supplemented with 10% FBS. Cells were cultured at 37°C in a humidified atmosphere containing 5% CO_2_. Control cultures received an equivalent amount of DMSO only. Bone marrow mononuclear cells (BMMCs) or mobilized peripheral blood mononuclear cells (PBMCs) were obtained from 10 newly diagnosed AML patients and 3 healthy donors. All donors provided written informed consent, and the study had the approval of the Institute Research Ethics Committee at Sun Yat-Sen University, in accordance with the Declaration of Helsinki. Patient characteristics are shown in Table 1. PBMCs and BMMCs were enriched from the diagnostic bone marrow samples of patients with de novo AML by Ficoll-Hypaque (Sigma-Aldrich, St Louis, MO) density gradient centrifugation.

**Table 1:** Characteristic of patients

Patient #	Age (Years)	Gender	FAB classification	WBC(*10^9^/L)	Source
P1	52	Male	M_0_	10.8	BM
P2	41	Male	M_5_	13.1	BM
P3	38	Male	M_3_	101.2	BM
P4	17	Male	M_3_	18.3	BM
P5	25	Female	M_5_	16.8	BM
P6	38	Female	M_3b_	103.6	BM
P7	71	Male	M_2b_	35.2	BM
P8	35	Female	M_3_	108.2	PB
P9	55	Male	M_2_	32.8	PB
P10	37	Male	M_2a_	5.2	PB

P, patient; FAB classification, French-American-British classification; WBC, white blood cells; BM, bone marrow; PB, peripheral blood.

### MTT assay

Cell viability was assessed by standard MTT assay (Sigma-Aldrich). The absorbance was determined at a test wavelength of 490 nm on a multiwell plate reader (Microplate Reader; Bio-Rad, Hercules, CA). Percent cell viability was calculated as cell viability of the experimental cells divided by cell viability of the control samples, times 100. At least three independent experiments were performed.

### Annexin V analysis

An Annexin V assay was used according to the manufacturer's instructions (Annexin V-FITC Apoptosis Detection Kit, EMD Biosciences). Briefly, approximately 5 × 10^5^/mL cells in 35 mm plates were treated with various concentrations of the indicated compounds. The cells were harvested and used for Annexin V-FITC/PI staining. The percentage of apoptotic cells was determined using FACS flow cytometer equipped with CellQuest software (BD Immunocytometry Systems).

### Cell cycle analysis

For the induction of cell cycle arrest and apoptosis by Ox-1, ZM, and/or APT-263, approximately 5 × 10^5^/mL cells in 6-well plates were treated with various concentrations of compounds for 48h. Single-cell suspensions were collected and fixed in ice-cold 70% ethanol overnight, labeled with 500 μL propidium iodide (50 μL/mL; Sigma-Aldrich) for at least 15 min in dark at room temperature (RT), and analyzed directly on a Beckon Dickinson FACScan (Oxford). The Sub G_1_ peak was utilized as a measure of apoptosis.

### Cell synchronization

Cells were synchronized at the G_1_/S transition by standard double thymidine (Sigma-Aldrich) blocking method. Briefly, cells were blocked for 16h with thymidine, subsequently released in fresh media for 8h and exposed to thymidine for an additional 16h, then released again in fresh media. Cells were then incubated in the presence of vinblastine at 1h and subsequently treated by Ox-1, CDK inhibitors, or DMSO at 10h. Finally, cells were collected at the indicated time points.

### Western blot analysis

Total cellular proteins were isolated with lysis buffer (20 mM Tris, pH 7.5; 150 mM NaCl; 0.25% NP40; 2.5 mM sodium pyrophosphate; 1 mM EGTA, 1 mM EDTA; 1 mM b-glycerophosphate; 1 mM Na3VO4; 1 mM PMSF; 1 μg/mL leupeptin). Equal amounts of cell extract were subjected to electrophoresis in SDS-polyacrylamide gel and transferred to nitrocellulose membrane (Bio-Rad). The membrane was blocked and then incubated with glyceraldehyde-3-phosphate dehydrogenase (GAPDH; Ambion), MAD2 (Abcam), cyclinB1, p-Bcl-xL (Ser62), Bcl-2 (all from Santa cruz, CA), CDC2, p-CDC2, Bcl-xL, cleaved poly(ADP)ribose polymerase (PARP), and BubR1 antibodies (all from Cell Signaling) overnight at 4°C and incubated with a HRP conjugated anti-mouse or anti-rabbit secondary antibody at room temperature for 1h. The protein bands were visualized using an enhanced chemiluminescence reagent (Pierce Biotechnology, USA), according to the manufacturer's instructions.

### Immunofluorescence staining

NB4 cells were incubated with Ox-1 at 10 μM for 48h. Cells were fixed in cold methanol for 20 min at 4°C and permeabilized in 0.5% TritonX-100 in PBS at RT for 15 min. Then cells were incubated with 1% BSA for 1h at RT to block nonspecific binding before the primary antibody reaction. Slides were incubated with the primary antibody to a-tubulin at RT for 1h, followed by Alexa Flour 680 or FITC 488 conjugated antibody. Nuclei were stained with DAPI (1 μg/mL), and cells were visualized using a microscope (1000x, Olympus).

### Short interfering RNA (siRNA) transfection

The sequences of siRNAs targeting Bcl-xL and Bcl-2 were selected based upon published literature: Bcl-xL RNAi-A, CAGCUUGGAUGGCCACUUAUU; Bcl-xL RNAi-B, ACAAGGAGAUGCAGGUAUUUU [38] and Bcl-2 RNAi-A [39]; RNAi-B, GGGAGAUAGUGAUGAAGUAUU [33]. The sequence used for scrambled control siRNA was UUCUCCGAACGUGUCACGU. Transfection of siRNAs was carried out using Lipofectamine 2000 (Invitrogen) according to the manufacturer's protocol.

### IP pull-down assay

To immunoprecipitate endogenous proteins, whole cell extracts were pre-cleared with normal IgG-AC (Santa Cruz) followed by overnight incubation at 4°C with antibody against Cdc20 (Cell signalling) [40]. The beads were washed three times with lysis buffer, and the immunoprecipitation complexes were subjected to SDS-PAGE.

### Statistical analysis

Statistical analysis was performed using SPSS version 13.0 (SPSS Inc.). Student's *t*-test was used to make a statistical comparison between groups. The level of significance was set at *p* < 0.05. Both Calcusyn software (Biosoft, Ferguson, MO, USA) [27, 28] and Jin's formula [29] were used to evaluate the synergistic effects of drug combinations. Jin's formula is given as: Q = E_a+b_/(E_a_ + E_b_ – E_a_ × E_b_), where E_a+b_ represents the cell proliferation inhibition rate of the combined drugs, while E_a_ and E_b_ represent the rates for each drug respectively. A value of Q = 0.85-1.15 indicates a simple additive effect, while Q > 1.15 indicates synergism. Combination index (CI) plots were generated using CalcuSyn software. A value of CI < 1 indicates synergism.

## SUPPLEMENTARY MATERIAL FIGURES


