# Supplementary material for: Inhibition of Bcl-xL overcomes polyploidy resistance and leads to apoptotic cell death in acute myeloid leukemia cells
Source: Oncotarget. 2015 May 27;6(25):21557–71. doi: 10.18632/oncotarget.4306 (PMC4673286; doi:10.18632/oncotarget.4306)
Supplement: Supplementary file 1 [file oncotarget-06-21557-s001.pdf]

Inhibition of Bcl-xL overcomes polyploidy resistance and leads to apoptotic cell death in acute myeloid leukemia cells

Supplementary Material

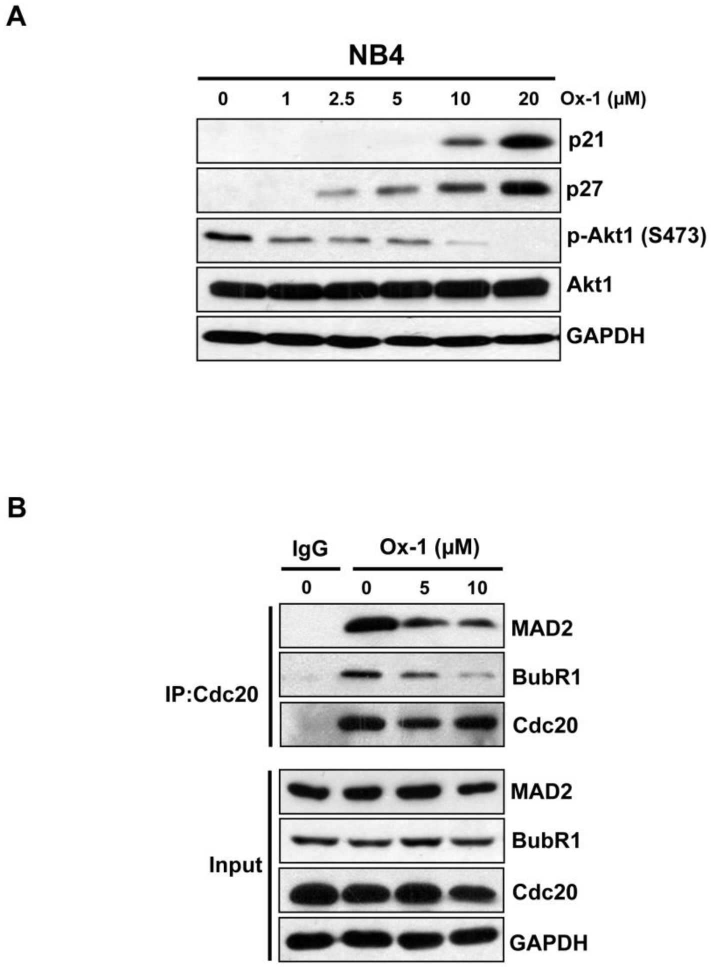

Figure S1. VEGFR or CDK4/cyclin D1 inhibitor Ox-1 induces SAC inactivation in AML cells. (A) Ox-1 targets VEGFR and CDK4/cyclin D1 in AML cell line. NB4 cells were treated with different doses of Ox-1 for 48h. Cells were then collected for the immunoblotting with indicated antibodies. **(B)** Ox-1 treatment decreases the binding between Cdc20 and MAD2 or BubR1. NB4 cells were treated with DMSO or Ox-1 for 48h prior to harvesting. Whole cell extracts were immunoprecipitated with antibodies against Cdc20, along with IgG control, followed by immunoblotting with indicated antibodies.

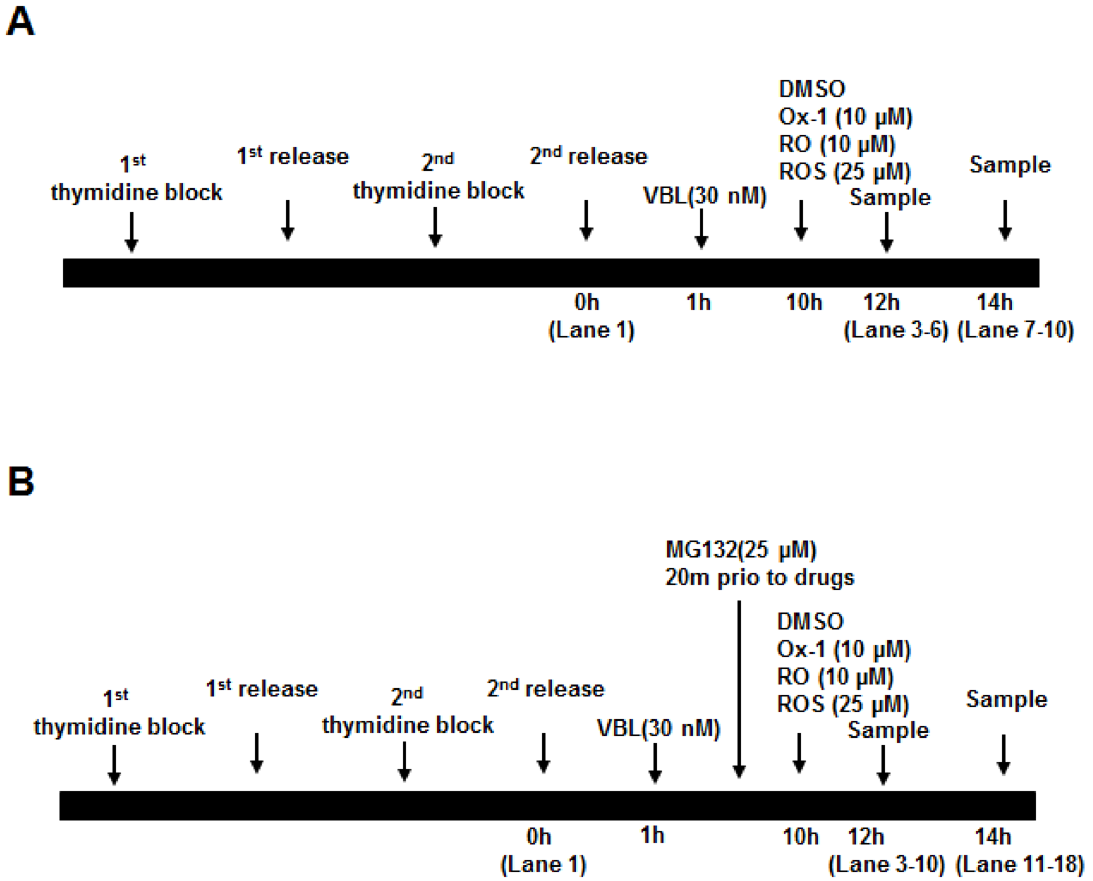

Figure S2. Experimental designs. (A) & **(B)** The timing of events for cell synchrony by a double thymidine block and cell cycle-specific timing of Ox-1 and CDK1 inhibition following vinblastine treatment are shown.

A

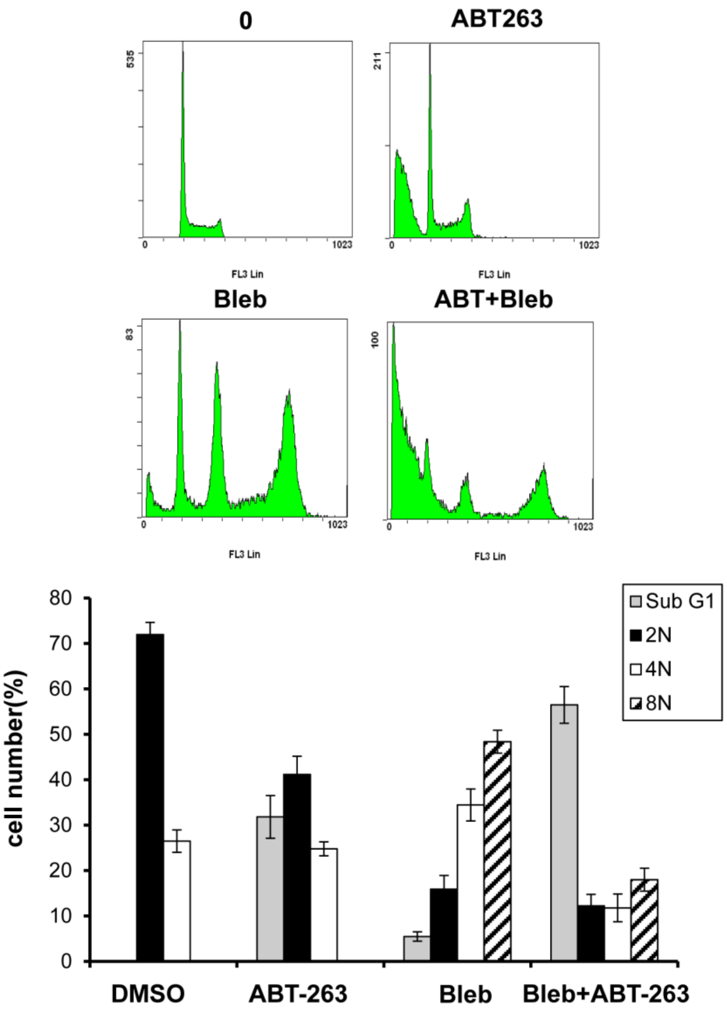

B

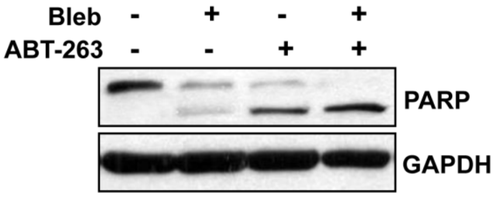

Figure S3. ABT-263 elicits synergistic cytotoxicity with the myosin II inhibitor blebbistatin. **(A)** ABT-263 blocks Ox-1-induced polyploidy. NB4 cells were incubated with DMSO, blebbistatin (25 µM), and/or ABT-263 (1 µM) for 48h; cells were then collected, fixed with ice-cold 70% ethanol overnight, and collected for propidium iodide staining and flow cytometry. Shown are mean ± SD, $^{**}P < 0.01$. **(B)** ABT-263 triggers apoptosis in Ox-1-induced polyploidy cells. NB4 cells were treated with DMSO, blebbistatin (25 µM), and/or ABT-263 (1 µM) for 48h; cells were then harvested for immunoblotting with indicated antibodies.

A

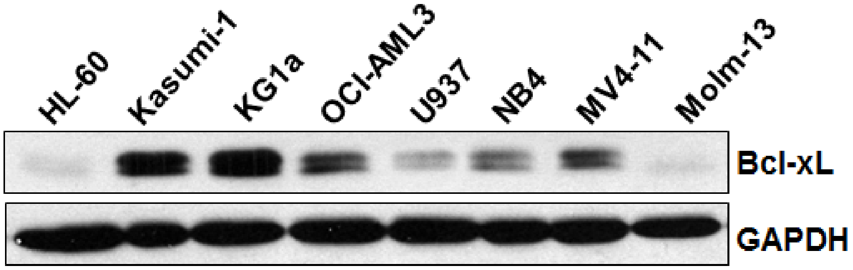

B

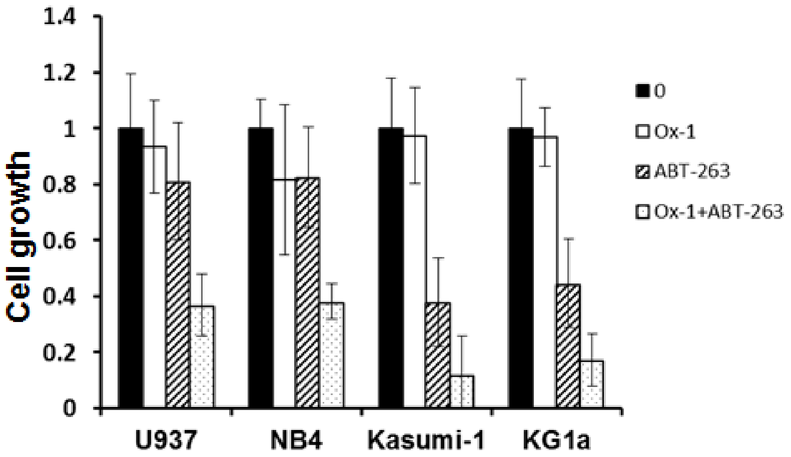

Figure S4. ABT-263 shows more synergistic inhibition of cell growth with Ox-1 in Bcl-xL overexpression cell lines. (A) Expression of Bcl-xL in multiple AML cell lines. **(B)** ABT-263 shows more synergistic inhibition of cell growth with Ox-1 in Kasumi-1 and KG1a cells than that in U937 and NB4 cells. Cells (5000 cells/well) were treated with DMSO or different doses of Ox-1 for 48h. MTT assay was performed to detect the cell viability
